# From Horizontal Root Fracture to Implant Rehabilitation: A Case Report

**DOI:** 10.7759/cureus.111933

**Published:** 2026-07-02

**Authors:** Rutuja B Salunkhe, N.D. Shashikiran, Namrata Gaonkar, Ruchira R Sawant, Sandisha S Sudrik

**Affiliations:** 1 Department of Pediatric and Preventive Dentistry, School of Dental Sciences, Krishna Vishwa Vidyapeeth (Deemed to be University), Karad, IND

**Keywords:** alveolar ridge preservation, crown-root fracture, dental implant, guided bone regeneration, maxillary central incisor, osseointegration, platelet-rich fibrin, socket preservation, sticky bone, trauma

## Abstract

Traumatic crown-root fractures involving previously endodontically treated anterior teeth can present substantial restorative difficulties, particularly when the fracture line extends below the alveolar crest and compromises the remaining tooth structure. Preservation of the alveolar ridge following extraction is essential to facilitate future implant-supported rehabilitation and optimize aesthetic outcomes. This report describes the management of a 17-year-old male who presented with pain and mobility of the maxillary right central incisor following dental trauma. The tooth had undergone prior root canal treatment but lacked a definitive coronal restoration. Clinical and radiographic evaluation revealed a horizontal crown-root fracture extending subcrestally, accompanied by a defect in the labial cortical plate. Owing to the unfavorable restorative prognosis, limited residual tooth structure, and inability to establish a predictable ferrule, the tooth was considered nonrestorable and extracted atraumatically. Socket preservation was performed using sticky bone prepared from injectable platelet-rich fibrin, advanced platelet-rich fibrin, and xenograft particles, followed by provisional rehabilitation with a Maryland bridge. Radiographic assessment after three months demonstrated adequate preservation of ridge architecture, permitting placement of a 3.5 × 13 mm dental implant with simultaneous guided bone regeneration. Definitive prosthetic rehabilitation was subsequently completed. This case highlights the potential benefit of combining autologous platelet concentrates with xenograft material for ridge preservation after the extraction of nonrestorable traumatized teeth, thereby supporting predictable implant placement and favorable functional and aesthetic outcomes in adolescent patients.

## Introduction

Traumatic dental injuries are frequently encountered in children and adolescents, with the maxillary central incisors being the most commonly affected teeth because of their anterior position within the dental arch [1]. The spectrum of injury ranges from uncomplicated enamel fractures to complex crown-root and root fractures that may significantly compromise function, aesthetics, and long-term tooth survival [2].

Management of crown-root fractures becomes particularly challenging when the fracture line extends below the alveolar crest. In such situations, the restorative prognosis is influenced by multiple factors, including the extent of the remaining tooth structure, ferrule availability, periodontal support, crown-root ratio, and the ability to maintain the supracrestal tissue attachment apparatus [3]. Deep subgingival fractures often result in biologic width violation, compromised isolation, and inadequate bonding surfaces, thereby reducing the predictability of restorative procedures and increasing the risk of future failure [4].

When a tooth is considered nonrestorable, extraction followed by implant-supported rehabilitation is frequently considered the most predictable treatment option. However, physiologic remodeling of the extraction socket results in substantial horizontal and vertical alveolar bone loss, particularly within the first few months after extraction [5]. These dimensional changes may complicate implant placement and negatively influence aesthetic outcomes in the anterior maxilla [6].

To minimize postextraction ridge resorption, various alveolar ridge preservation techniques have been advocated. Systematic reviews have demonstrated that socket preservation procedures significantly reduce ridge dimensional changes compared with extraction alone [7]. In recent years, autologous platelet concentrates have gained considerable attention because of their ability to enhance wound healing and promote tissue regeneration through the sustained release of growth factors [8].

Injectable platelet-rich fibrin (I-PRF) and advanced platelet-rich fibrin (A-PRF) can be combined with particulate graft materials to produce a biologically active composite known as sticky bone. This grafting approach provides improved handling characteristics, graft stability, and a favorable microenvironment for angiogenesis and bone regeneration [9]. Such regenerative strategies may be particularly advantageous in traumatic defects located within the aesthetic zone, where preservation of hard and soft tissue architecture is essential for successful implant rehabilitation.

The present case report describes the multidisciplinary management of a nonrestorable horizontal crown-root fracture of a maxillary central incisor using socket preservation with sticky bone, followed by delayed implant placement and guided bone regeneration, resulting in satisfactory functional and aesthetic rehabilitation.

## Case presentation

A 17-year-old male presented to the Department of Pediatric and Preventive Dentistry with pain, mobility, and aesthetic concerns involving the maxillary right central incisor (tooth 11). The patient reported a history of trauma two days before presentation. The affected tooth had undergone root canal treatment several months earlier following a previous traumatic episode; however, the patient had failed to return for definitive full-coverage crown rehabilitation (Figure 1).

**Figure 1:**
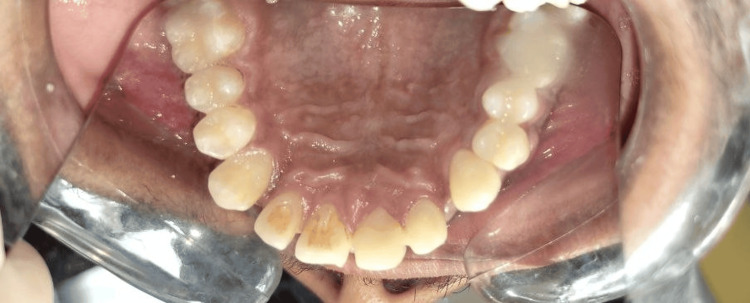
Clinical examination findings showing a fracture line on tooth 21

Radiographic examination revealed a horizontally fractured maxillary right central incisor with Grade II mobility of the coronal segment (Figure 2).

**Figure 2:**
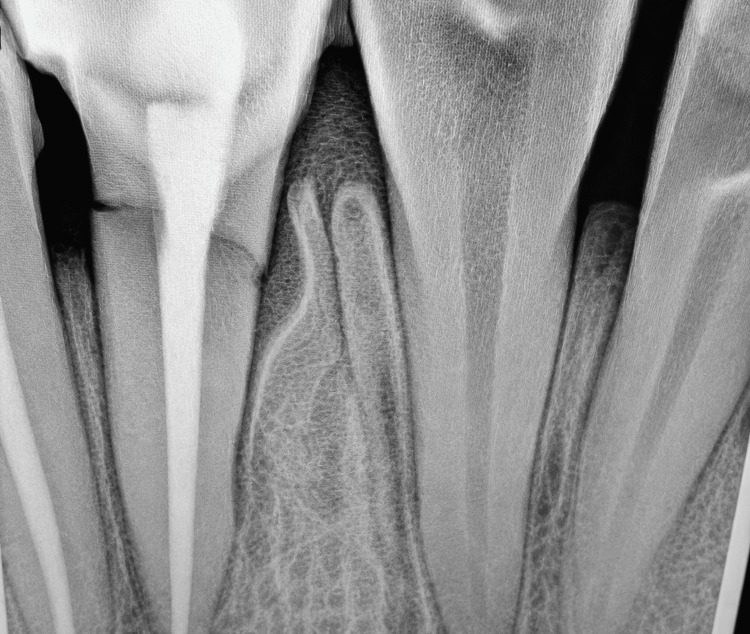
Radiographic examination revealing a horizontally fractured maxillary right central incisor

The tooth was tender on percussion and associated with localized gingival inflammation. A gentle mobility stress test confirmed instability of the coronal fragment. The fracture line extended subgingivally, raising concerns regarding the long-term restorative prognosis.

Cone-beam CT (CBCT) was performed for comprehensive assessment. The scan demonstrated a horizontal crown-root fracture extending below the alveolar crest with associated loss of labial bony support (Figure 3).

**Figure 3:**
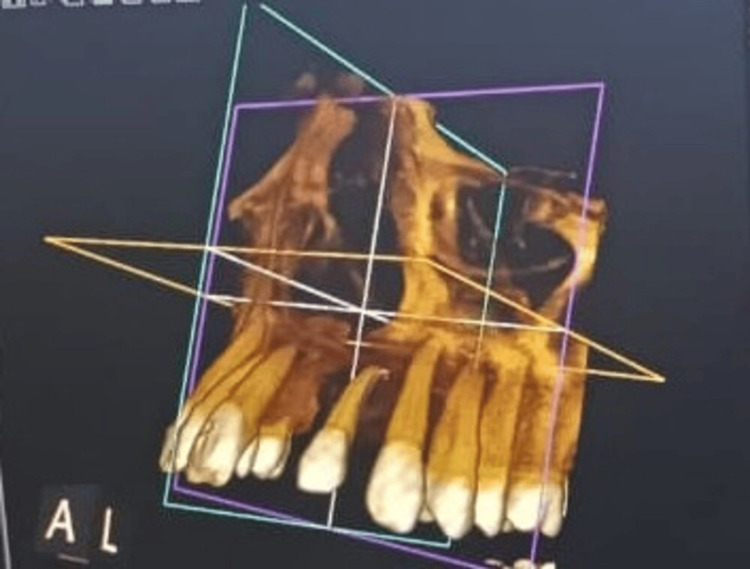
CBCT scan demonstrating a horizontal crown-root fracture extending below the alveolar crest CBCT, cone-beam CT

The remaining coronal tooth structure was insufficient to establish an adequate ferrule effect, and the subcrestal extension of the fracture compromised the biologic width. Considering the inadequate remaining tooth structure, poor restorative prognosis, unfavorable crown-root ratio, and anticipated risk of recurrent restorative failure, the tooth was deemed nonrestorable (Figure 4).

**Figure 4:**
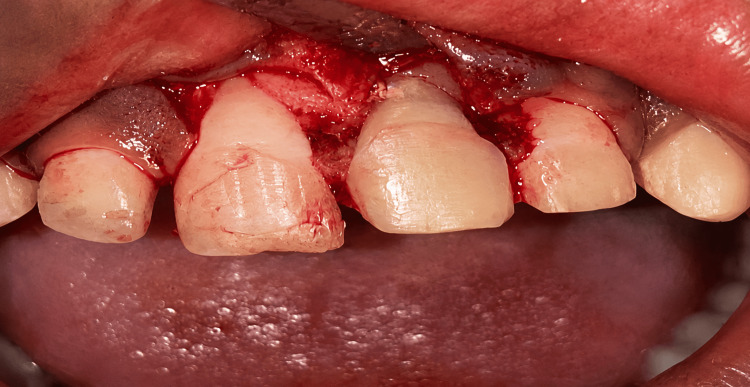
Clinical examination showing subcrestal extension of the fracture

As an emergency stabilization measure, a flexible splint using 0.4-mm (26-gauge) orthodontic wire and composite resin was applied (Figure 5).

**Figure 5:**
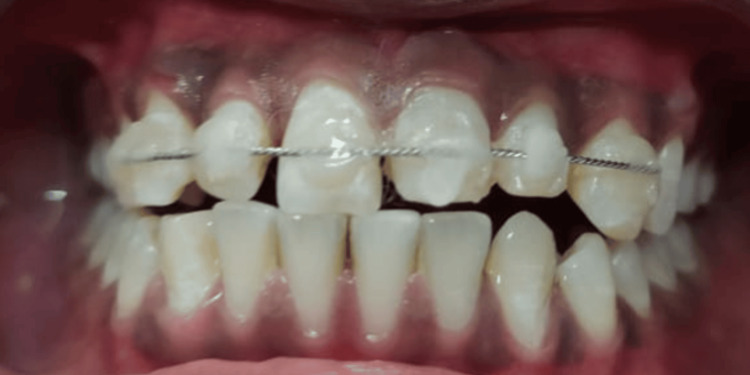
Splinting performed using 0.4-mm (26-gauge) orthodontic wire and composite resin

Following a detailed discussion of available treatment options, extraction with alveolar ridge preservation and delayed implant placement was selected as the definitive treatment approach. Immediate implant placement was not considered because of the presence of a labial bone defect and concerns regarding primary implant stability and aesthetic predictability. Under local anesthesia using 2% lignocaine, vertical releasing and crevicular incisions were placed, and a full-thickness mucoperiosteal flap was reflected (Figure 6).

**Figure 6:**
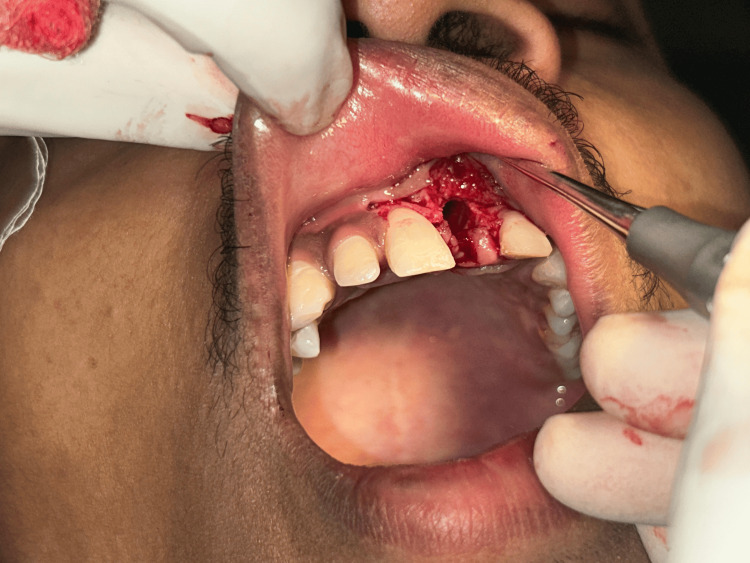
Full-thickness mucoperiosteal flap being reflected and the tooth being extracted

The fractured tooth was extracted atraumatically to preserve the remaining socket walls (Figure 7).

**Figure 7:**
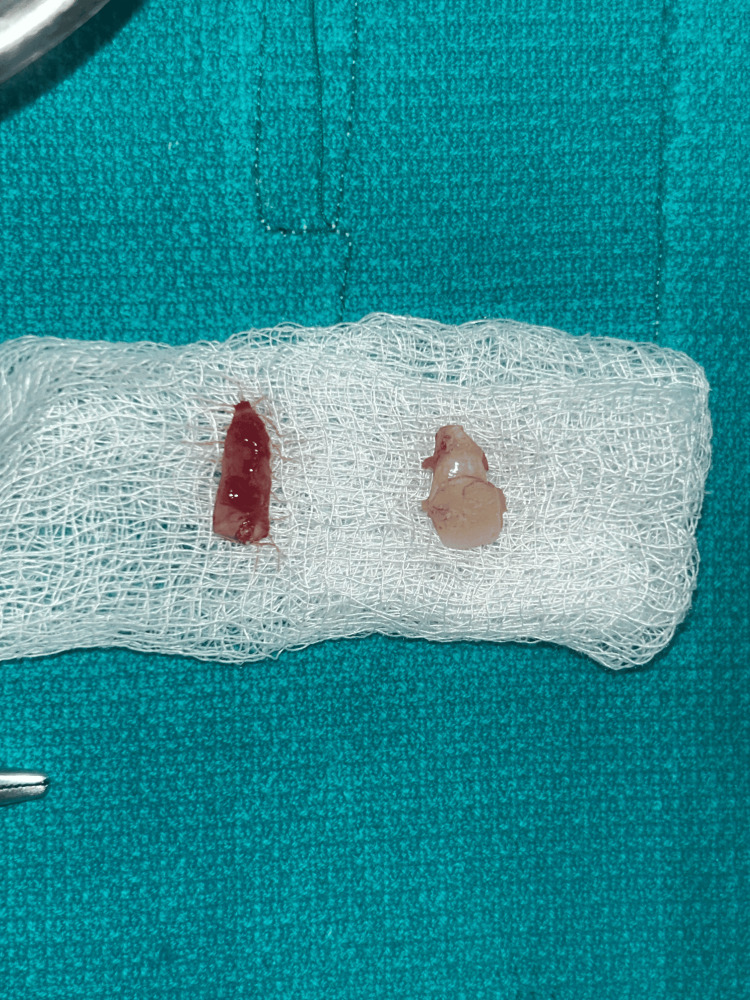
Extracted tooth

Ten milliliters of autologous venous blood were collected immediately before surgery and divided into two tubes. I-PRF was prepared by centrifugation at 700 rpm for three minutes, while A-PRF was obtained by centrifugation at 3000 rpm for 10 minutes. The prepared I-PRF and A-PRF were mixed with xenograft particles to form a cohesive sticky bone graft [8]. The graft material, Osseograft™ (DMBM-Xenograft, Advanced Biotech Products (P) Ltd., Chennai, India), a sterile, bioresorbable demineralized bone matrix xenograft with a particle size of approximately 250 μm, was placed into the extraction socket to promote ridge preservation and facilitate future implant rehabilitation (Figure 8).

**Figure 8:**
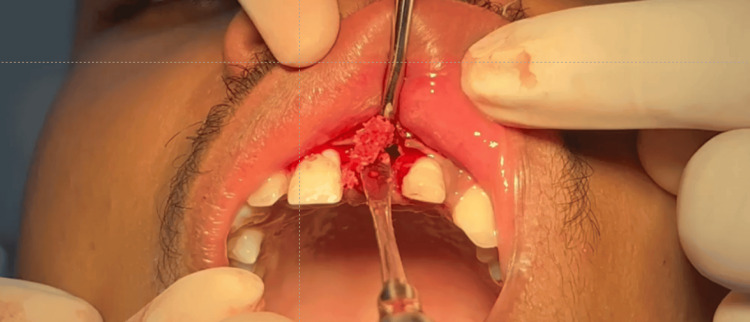
Placement of sticky bone graft material into the extraction socket

Primary closure was achieved, and postoperative instructions were provided. At the one-week review, satisfactory soft tissue healing was observed (Figure 9).

**Figure 9:**
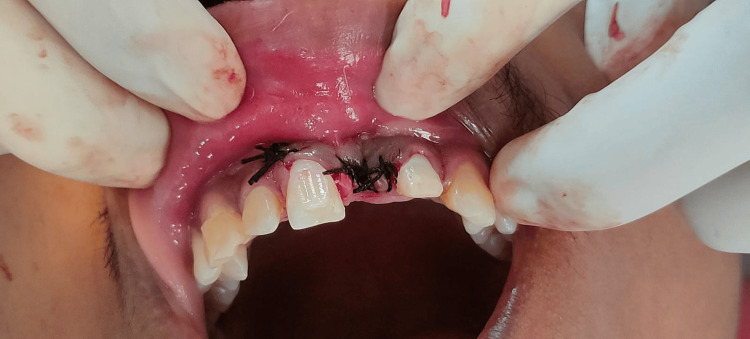
Suture placement and achievement of primary closure

An interim Maryland bridge was fabricated, providing a minimally invasive interim solution until definitive implant rehabilitation (Figure 10).

**Figure 10:**
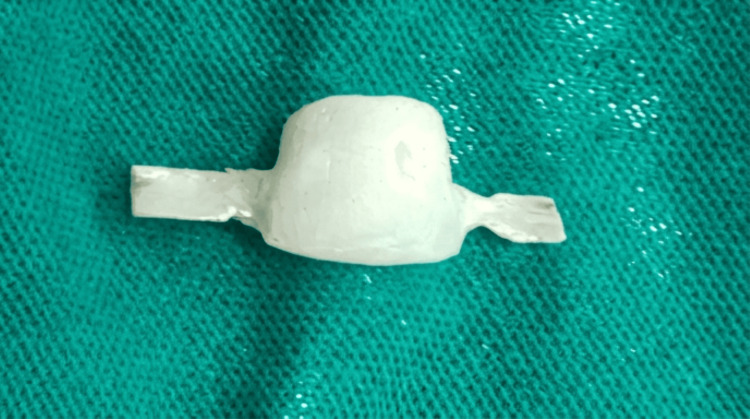
Fabrication of the Maryland bridge

A Maryland bridge was delivered to restore aesthetics and maintain the edentulous space during healing. The prosthesis was adhesively bonded to the adjacent teeth (Figure 11).

**Figure 11:**
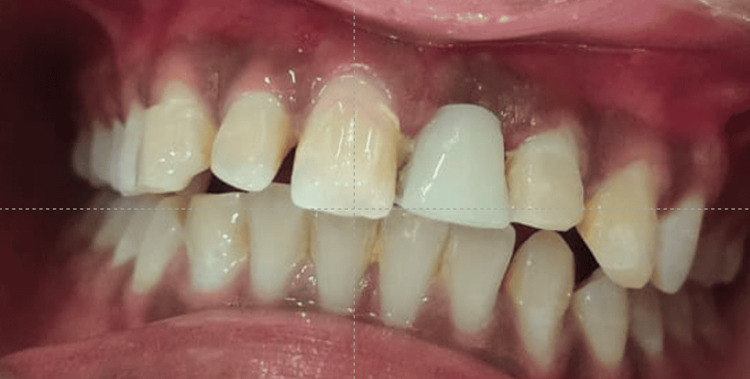
Delivery of the Maryland bridge

Three months after socket preservation, CBCT evaluation demonstrated a reduction in the labial defect height from 7.5 mm to 1.2 mm and the defect width from 4.8 mm to 0.8 mm. The ridge width increased from 4.2 mm to 7.1 mm, with a horizontal bone gain of approximately 2.9 mm. Ridge height was maintained at approximately 14 mm, suitable for implant placement. Axial and sagittal localizations in the anterior maxilla are shown in Figure 12A. Detailed linear measurements of the nasopalatine canal are shown in Figure 12B.

**Figure 12:**
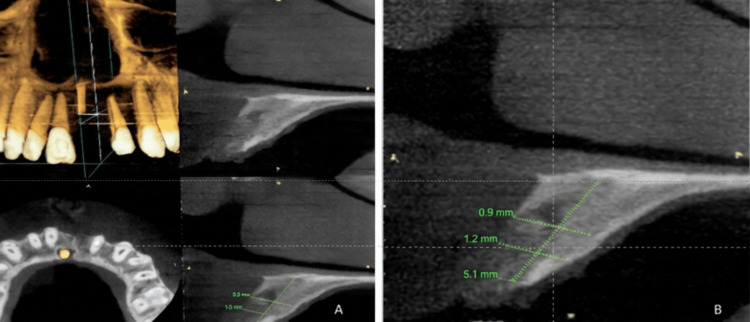
CBCT examination showing the selection of a 3.5 × 13 mm implant (A) Axial and sagittal localizations in the anterior maxilla. (B) Detailed linear measurements of the nasopalatine canal. CBCT, cone-beam CT

Following informed consent, implant surgery was performed under local anesthesia. A full-thickness flap was elevated using crevicular and vertical releasing incisions. A dental implant measuring 3.5 mm × 13 mm was inserted in the planned prosthetic position (Figure 13A). Successful placement of the dental implant at the surgical site before flap closure was achieved. Primary implant stability was achieved with an insertion torque of 35 N·cm (Figure 13B).

**Figure 13:**
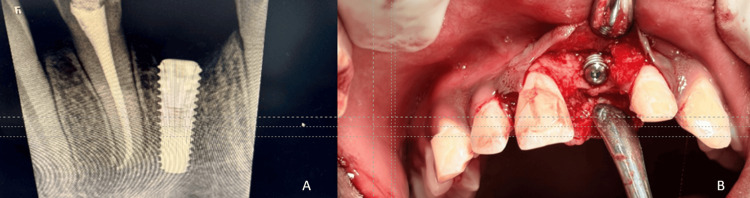
Implant placement (A) Post-placement periapical radiograph of the dental implant. (B) Corresponding clinical view of the implant surgical site before flap closure.

Guided bone regeneration was performed using a barrier membrane adapted to the defect morphology (Figure 14A). The barrier membrane was closely adapted to the defect morphology (Figure 14B). The bone graft was then placed (Figure 14C), and the flap was repositioned and secured with interrupted sutures to obtain tension-free primary closure (Figure 14D).

**Figure 14:**
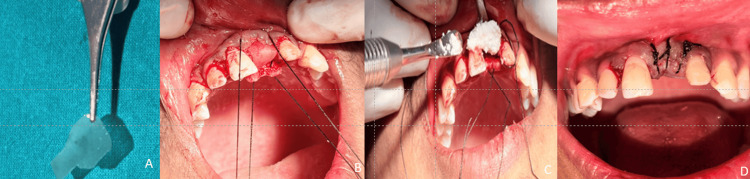
Step-by-step bone regeneration procedure (A) Guided bone regeneration. (B) Adaptation of the barrier membrane to the defect morphology. (C) Bone graft placement. (D) Flap repositioning and securement with interrupted sutures to obtain tension-free primary closure.

At the three-month follow-up, the labial defect height decreased from approximately 7.2 mm to 0.8 mm, and the labial defect width decreased from 4.6 mm to 0.6 mm. The ridge width increased from 4.1 mm preoperatively to 7.2 mm postoperatively, while ridge height was maintained from 13.5 mm to 14.3 mm, providing adequate bone volume and primary stability for placement of a 3.5 × 13 mm dental implant. The implant remained stable with healthy peri-implant soft tissues.

## Discussion

Management of traumatic crown-root fractures in the anterior maxilla remains one of the most challenging situations in restorative and implant dentistry. Treatment planning requires careful assessment of fracture extent, periodontal involvement, remaining tooth structure, aesthetic demands, and long-term prognosis. Although conservative approaches such as fragment reattachment, orthodontic extrusion, surgical crown lengthening, and post-core rehabilitation have been described, their success is highly dependent on the availability of sound tooth structure and the ability to establish an adequate ferrule effect [10].

In the present case, the fracture extended below the alveolar crest and involved an endodontically treated maxillary central incisor. CBCT examination revealed loss of labial bony support in addition to the subcrestal fracture extension. These findings significantly reduced the predictability of restorative treatment. Previous studies have demonstrated that the ferrule effect is a critical determinant of survival in endodontically treated teeth. Inadequate ferrule height increases stress concentration at the cervical region and predisposes the tooth-restoration complex to biomechanical failure [3]. Consequently, attempts to retain severely compromised teeth may result in repeated restorative failures, progressive periodontal breakdown, and eventual loss of additional supporting bone.

The decision to extract rather than pursue restorative salvage was based on the overall structural prognosis of the tooth. Preservation of a severely compromised tooth is not always synonymous with long-term success. Contemporary treatment planning increasingly emphasizes preservation of alveolar architecture and future restorative predictability rather than short-term retention of a structurally unsound tooth [11]. In the present case, extraction was considered the most biologically and mechanically favorable option for long-term rehabilitation.

A major concern following extraction in the aesthetic zone is the dimensional alteration of the alveolar ridge. Postextraction remodeling begins immediately and is most pronounced during the first three to six months. Systematic reviews have consistently demonstrated substantial horizontal and vertical bone reduction after extraction, particularly affecting the facial plate of the anterior maxilla [5,12]. Such dimensional changes may compromise implant positioning, soft-tissue support, and aesthetic outcomes.

To counteract these changes, socket preservation was performed using a sticky bone graft composed of xenograft particles combined with I-PRF and A-PRF. The biologic rationale for this approach lies in the ability of platelet concentrates to provide a sustained release of growth factors, including platelet-derived growth factor, transforming growth factor beta, vascular endothelial growth factor, and insulin-like growth factor, all of which contribute to angiogenesis and tissue regeneration [13]. Unlike conventional platelet-rich plasma, platelet-rich fibrin is prepared without anticoagulants and forms a fibrin matrix capable of gradually releasing bioactive molecules over time [14].

The incorporation of I-PRF into particulate graft materials results in the formation of sticky bone, a cohesive grafting complex that improves handling characteristics and graft stability. Enhanced graft stabilization may reduce particle migration and improve maintenance of the regenerative space during healing [15]. Furthermore, the fibrin network acts as a biologically active scaffold that supports cellular migration and vascular ingrowth. These characteristics are particularly beneficial in traumatic extraction sockets, where preservation of ridge dimensions is essential for future implant rehabilitation.

A-PRF was additionally utilized because low-speed centrifugation protocols have been associated with increased cellular content and enhanced release of growth factors compared with earlier platelet concentrate formulations [16]. Experimental investigations have demonstrated that A-PRF contains higher concentrations of leukocytes and regenerative cells capable of supporting tissue repair and bone regeneration [17]. Although the precise contribution of platelet concentrates to bone formation remains an area of ongoing investigation, accumulating evidence supports their adjunctive role in regenerative procedures.

Immediate implant placement was not selected in the present case because CBCT evaluation demonstrated a labial osseous defect and compromised socket morphology. Predictable immediate implant placement requires adequate residual socket walls and the ability to achieve primary stability in an ideal restorative position. In sockets with facial bone deficiencies, immediate placement may increase the risk of soft tissue collapse, peri-implant recession, and aesthetic complications [18]. Delayed implant placement following ridge preservation therefore represented a more predictable strategy for achieving optimal hard- and soft-tissue outcomes.

Three months after socket preservation, radiographic evaluation demonstrated maintenance of ridge dimensions sufficient for implant placement. Guided bone regeneration was performed simultaneously with implant insertion to further optimize peri-implant bone support. Successful osseointegration and favorable peri-implant tissue health were observed during follow-up, supporting the effectiveness of the staged treatment approach [19,20].

The present report highlights the value of combining biologically enhanced ridge preservation with delayed implant rehabilitation in the management of nonrestorable traumatic anterior tooth fractures. Implant placement in adolescents should be considered only after careful evaluation because residual craniofacial growth may influence long-term aesthetic outcomes. In the present case, the tooth was deemed nonrestorable, and delayed implant placement was undertaken only after successful socket preservation had established adequate hard and soft tissue architecture. The patient was 17 years old, close to the expected completion of facial skeletal growth, and the treatment decision was based on the clinical need for definitive rehabilitation, with planned periodic clinical and radiographic follow-up to monitor implant position and peri-implant tissues. Although longer follow-up is necessary to evaluate long-term peri-implant stability, the short-term clinical and radiographic outcomes suggest that the use of sticky bone may provide a predictable foundation for implant-supported rehabilitation in challenging aesthetic-zone defects.

## Conclusions

Management of traumatic crown-root fractures in endodontically treated anterior teeth requires a comprehensive evaluation of restorability, periodontal support, and long-term prognosis. In the present case, the subcrestal extension of the horizontal fracture, inadequate remaining tooth structure, compromised ferrule potential, and associated labial bone defect rendered the maxillary central incisor nonrestorable. A staged treatment approach involving atraumatic extraction, socket preservation with sticky bone prepared from I-PRF, A-PRF, and xenograft, followed by delayed implant placement with guided bone regeneration, resulted in successful rehabilitation of the affected site.

The use of biologically active autologous platelet concentrates contributed to the maintenance of ridge dimensions and provided a favorable foundation for subsequent implant therapy in the aesthetic zone. A three-month follow-up demonstrated satisfactory healing, implant stability, and radiographic evidence of osseointegration, with restoration of function and aesthetics. This case highlights the importance of preserving alveolar ridge architecture when retention of a traumatized tooth is no longer feasible. Sticky bone-assisted socket preservation, combined with careful implant planning and guided bone regeneration, may represent a predictable treatment option for managing nonrestorable traumatic anterior tooth fractures in young patients. The novelty of this case lies in the staged regenerative rehabilitation of a previously endodontically treated, horizontally fractured maxillary central incisor with a compromised labial plate in an adolescent patient. A sequential protocol incorporating sticky bone-assisted socket preservation followed by delayed implant placement with simultaneous guided bone regeneration successfully restored the deficient implant site and enabled predictable aesthetic rehabilitation.
